# Relationship between sodium–glucose cotransporter-2 inhibitors and muscle atrophy in patients with type 2 diabetes mellitus: a systematic review and meta-analysis

**DOI:** 10.3389/fendo.2023.1220516

**Published:** 2023-09-15

**Authors:** Chengdong Xia, Yufeng Han, Chunhui Yin, Ruyue Geng, Zhenfei Liu, Yongle Du, Mingkun Yu

**Affiliations:** ^1^ Department of Endocrinology, Xiyuan Hospital of China Academy of Chinese Medical Sciences, Beijing, China; ^2^ Shandong University of Traditional Chinese Medicine, Jinan, China; ^3^ Shandong First Medical University, Jinan, China

**Keywords:** SGLT-2i, muscle atrophy, type 2 diabetes mellitus, systematic review, meta-analysis

## Abstract

**Aim:**

This study aims to assess the association between sodium–glucose cotransporter type-2 inhibitor (SGLT-2i) treatment and muscle atrophy in patients with type 2 diabetes mellitus (T2DM).

**Methods:**

We searched six databases from 1 January 2012 to 1 May 2023, without language restrictions. The primary outcome was muscle. Secondary outcomes were weight loss, weakness, malaise, or fatigue. Subgroup analyses were performed according to different definitions of muscle, treatment duration, and measurement methods. The quality of the studies was assessed using the Cochrane tool. The quality of the evidence was assessed using the Grading of Recommendations, Assessment, Development and Evaluations (GRADE) tool.

**Results:**

Nineteen randomized controlled trials (RCTs) involving 1,482 participants were included. Compared with the control group, a meta-analysis showed that T2DM participants in the group treated with SGLT-2i demonstrated statistically significant reductions in lean body mass of 0.66 (95% confidence interval (CI), −1.05 to −0.27; *p* = 0.0009) and skeletal muscle mass of 0.35 (95% CI, −0.66 to −0.04; *p* = 0.03). No deaths or serious adverse events were reported. The quality of evidence in the included trials was low.

**Conclusions:**

SGLT-2i may lead to a reduction in muscle strength in the treatment of T2DM compared to the control group. However, there is still a lack of high-quality evidence to evaluate muscle atrophy caused by SGLT-2i.

**Systematic review registration:**

https://inplasy.com/inplasy-2022-12-0061/, identifier 2022120061.

## Introduction

The global incidence of type 2 diabetes mellitus (T2DM) is very high, and the International Diabetes Federation (IDF) estimates that approximately 463 million people suffered from T2DM in 2019. This number will reach 700 million by the year 2045 (1). T2DM and its complications will contribute substantially to the global rates of death and disability. Commonly used treatments include lifestyle interventions, pharmacological interventions, and bariatric surgery (2).

Sodium-glucose cotransporter-2 inhibitors (SGLT-2i) represent a new type of oral hypoglycemic agent (3). The world’s first SGLT-2i, dapagliflozin, was approved in 2012 for treating T2DM in Europe, with nearly 10 types of SGLT-2i in clinical use up to the present (4). The incidence and mortality from T2DM are considered to be mainly related to chronic cardiovascular complications. SGLT-2i lowers blood glucose mainly by promoting urinary glucose excretion and can assist in reducing weight, blood pressure, and cardiovascular and renal risks (5–9). The pathophysiology of the cardioprotective outcome produced by the application of SGLT-2i is not known. Possible mechanisms of action include its role in anti-inflammatory and oxidative stress pathways (10). A recent review summarized animal studies and clinical trials of SGLT-2i analogs, which showed that they have pharmacological effects in the treatment of diabetes by attenuating oxidative stress associated with T2DM, which may explain their cardiovascular benefits and have promising applications in the treatment of T2DM (11). A previous large cardiovascular outcomes trial investigated the efficacy of empagliflozin in 7,020 patients with T2DM and established ASCVD and reported a significant 38% reduction in cardiovascular mortality and a 32% reduction in heart failure. This review not only mentioned empagliflozin but also mentioned the pharmacological effects of other SGLT-2i on diabetes (11). Their pharmacological effects are similar. Standards of Medical Care in Diabetes-2021 stated that among patients with type 2 diabetes who have established atherosclerotic cardiovascular disease (ASCVD) or indicators of high risk, established kidney disease, or heart failure, a SGLT-2i is recommended as part of a glucose-lowering regimen independent of A1C and in consideration of patient-specific factors (12). Additionally, the latest guidelines of the American Diabetes Association (ADA) recommend that SGLT-2i be actively recommended to all patients with T2DM who also have comorbid cardiovascular disease (13). As can be seen from the above, SGLT-2i has gained great importance in the treatment of type 2 diabetes patients with cardiovascular and other diseases.

Symptoms of muscle atrophy and weakness were observed during T2DM treatments with SGLT-2i. Some patients experience improvement or complete regression of these symptoms after discontinuing SGLT-2i (14–17). Additional case reports suggest a potential relationship between SGLT-2i use and myopathy episodes, both of which raise concerns about SGLT-2i-induced muscle atrophy. However, current clinical trials involving the effects of SGLT-2i on muscles have shown inconsistent results. Several clinical trials have shown beneficial or insubstantial effects of SGLT-2i application on muscle (18, 19), whereas others have reached the opposite conclusion (20). Overall, the actual effect of SGLT-2i on muscles is not known.

The aim of this systematic review and meta-analysis was to evaluate the association between SGLT-2i and muscle atrophy when treating patients with T2DM, with the aim of providing a basis for clinical practice and new directions for research in this field.

## Methods

This study followed the Preferred Reporting Items for Systematic Reviews and Meta-analyses (PRISMA) reporting guidelines (21, 22). The protocol was registered in INPLASY (2022120061). The DOI number is 10.37766/inplasy2022.12.0061.

### Search strategy

We searched six databases, including PubMed, Web of Science, Embase, Cochrane Library, Chinese National Knowledge Infrastructure (CNKI), and Wan Fang Database, for randomized controlled trials of SGLT-2i for T2DM from 1 January 2012 to 1 May 2023. A detailed search strategy is presented in **Supplementary Material 1**.

### Eligibility criteria

Studies were included in the systematic screening if they met the following criteria: (1) the study was conducted in adults with T2DM; (2) the use of SGLT-2i, including canagliflozin, dapagliflozin, empagliflozin, tofogliflozin, ipragliflozin, luseogliflozin, and so on, had been prescribed to patients with T2DM; (3) single or add-on therapy with SGLT-2i as an intervention, with no restrictions on dosage or frequency of use; (4) data on at least one of the outcome indicators of muscle mass, skeletal muscle mass, fat-free mass, or lean body mass was provided, with information on the mean and standard deviation of the change in the above outcome indicators; and (5) the design was a randomized controlled trial. Studies were excluded if they included (1) pregnant women, (2) adults without diabetes, (3) SGLT-2i treatment prior to the intervention, (4) combination formulations of SGLT-2i in fixed-dosage combinations with other commonly used drugs, (5) an intervention period of less than 4 weeks, (6) a lack of required outcome data, (7) nonrandomized controlled trials, and (8) duplicate reports.

### Study selection

The retrieved studies were imported into NoteExpress, and duplicate records were removed. The title, abstract, and full-text screening were independently evaluated by three reviewers (Chengdong Xia, Yufeng Han, and Chunhui Yin). Any disagreements between the three reviewers were resolved through discussion with a fourth reviewer or a senior author.

### Data extraction

**Table 1** lists the trial-level characteristics of the 19 studies, including the first author, year of publication, sample size, baseline characteristics of the participants (age, HbA1c, and body mass index), interventions and controls, treatment duration, and measurement method. The risk factors evaluated in this meta-analysis were the mean and standard deviation of the measures of change before and after the intervention. The definitions were largely similar (40). The data were extracted independently by three authors (Chengdong Xia, Yufeng Han, and Chunhui Yin). Disagreements were resolved through discussion with a fourth reviewer or senior author.

**Table 1:** Characteristics of the included 19 studies.

Studies	Sample (S; C)	Age, y(S; C)	HbA1c, %(S;C)	BMI, kg/m^2^ (S;C)	Interventions (S; C)	Measurement method	Treatment duration, w
McCrimmon (2020) (20)	S: 90	S: 58.6 (10.1)	S: 8.3 (1.0)	S: 32.3 (5.5)	S: Canagliflozin	DXA	52
	C: 88	C: 57.8 (9.9)	C: 8.5 (1.1)	C: 32.6 (6.4)	C: Semaglutide		
Han (2020) (23)	S: 30	S: 52.5 (10.3)	S: 6.7 (0.7)	S: 30.4 (5.4)	S: Ipragliflozin + metformin + pioglitazone	DXA	24
	C: 15	C: 56.7 (11.8)	C: 6.6 (0.6)	C: 30.2 (2.5)	C: Metformin + pioglitazone		
Inoue (2019) (24)	S: 22	51 + 9	S: 8.12 (0.93)	S: 27.9 (4.0)	S: Ipragliflozin + insulin	BIA	24
	C: 24		C: 8.3 (0.65)	C: 27.7 (4.5)	C: Insulin		
Horibe (2022) (25)	S: 26	S: 59.7 (12.0)	S: 7.68 (0.53)	S: 28.0 (4.0)	S: Dapagliflozin + OAD	DXA	24
	C: 24	C: 62.3 (6.5)	C: 7.73 (0.50)	C: 27.6 (3.8)	C: OAD		
Shimizu (2019) (26)	S: 33	S: 56.2 (11.5)	S: 8.37 (1.48)	S: 27.6 (4.7)	S: Dapagliflozin	BIA	24
	C: 24	C: 57.1 (13.8)	C: 7.7 (1.24)	C: 28.3 (3.5)	C: Standard treatment without SGLT2 inhibitors		
Yamakage (2020) (9)	S: 27	S: 58.4 (13.0)	S: 7.5 (0.8)	S: 31.3 (7.6)	S: Dapagliflozin	BIA	24
	C: 27	C: 60.7 (11.9)	C: 7.4 (0.9)	C: 30.7 (6.2)	C: Conventional medications		
Sugiyama (2018) (27)	S: 28	S: 55.6 (7.4)	S: 7.9	S: 27.5 (2.4)	S: Dapagliflozin	BIA	24
	C: 22	C: 56.7 (7.9)	C: 7.6	C: 26.2 (3.4)	C: Non-SGLT2i		
Kitazawa (2020) (28)	S: 32	20-75	S: 7.4 (0.5)	S: 25.3 (3.9)	S: Tofogliflzin + DPP4i + metformin	BIA	24
	C: 29		C: 7.5 (0.4)	C: 25.4 (3.8)	C: Glimepiride + DPP4i + metformin		
Fadini (2017) (29)	S: 15	S: 66.3 (1.8)	S: 8.2 (0.2)	S: 28.4 (1.4)	S: Dapagliflozin	BIA	12
	C: 16	C: 61.0 (1.8)	C: 8.2 (0.2)	C: 32.8 (1.4)	C: Placebo		
Kim (2014) (30)	S: 36	55.7	NA	NA	S: Empagliflozin + metformin	DXA	52
	C: 26				C: Glimepiride + metformin		
Wolf (2021) (31)	S: 44	S: 58 (7)	S: 7.7 (1.2)	S: 30 (7)	S: Dapagliflozin	DXA	12
	C45:	C: 58 (7)	C: 7.9 (1.4)	C: 31 (7)	C: Glibenclamide		
Nakaguchi (2020) (32)	S: 31	S: 66.3 (9.5)	S: 8.08 (0.76)	S: 25.8 (4.1)	S: Empagliflozin + insulin	DXA	24
	C: 30	C: 67.2 (9.0)	C: 8.04 (0.75)	C: 26.4 (4.6)	C: Liraglutide + insulin		
Cefalu (2013) (33)	S1: 71	S1: 56.4 (9.5)	S1: 7.8 (0.8)	S1: 31.0 (5.3)	S1: Canagliflozin 100 mg	DXA	52
	S2: 69	S2: 55.8 (9.2)	S2: 7.8 (0.8)	S2: 31.2 (5.4)	S2: Canagliflozin 300 mg		
	C: 68	C: 56.3 (9.0)	C: 7.8 (0.8)	C: 30.9 (5.5)	C: Glimepiride		
Blonde (2016) (34)	S1: 56	S1: 64.3 (6.6)	S1: 7.8 (0.8)	S1: 30.9 (4.8)	S1: Canagliflozin 100 mg	DXA	26
	S2: 60	S2: 63.0 (6.0)	S2: 7.8 (0.8)	S2: 31.6 (4.3)	S2: Canagliflozin 300 mg		
	C: 50	C: 64.2 (6.4)	C: 7.8 (0.7)	C: 32.0 (5.5)	C: Placebo		
Igarashi (2023) (35)	S: 8	S: 53.8 (15.2)	S: 11.8 (1.3)	S: 26.3 (5.1)	S: Canagliflozin + CON	InBody	12
	C: 8	C: 50.4 (14.0)	C: 12.9 (1.8)	C: 27.7 (5.1)	C: CON		
Bolinder (2014) (36)	S: 66	60.7	7.2	31.9	S: Dapagliflozin + metformin	DXA	102
	C: 71				C: Placebo + metformin		
Kang (2022) (37)	S: 63	20–75	S: 8.5 (0.7)	≥23	S: Ipragliflozin	InBody	24
	C: 67		C: 8.5 (0.7)		C: Sitagliptin		
Zeng (2022) (38)	S: 46	S: 58.9 (9.9)	S: 9.2 (1.4)	S: 27.7 (5.0)	S: Empaglifozin + premixed insulin	BIA	24
	C: 51	C: 58.7 (10.2)	C: 9.0 (1.2)	C: 28.0 (3.5)	C: Linagliptin + premixed insulin		
Kayano (2020) (39)	S: 36	S: 69.4 (7.1)	S: 7.6 (0.7)	S: 25.6 (4.2)	S: Traditional treatment + dapagliflozin	InnerScan V with reactance technology	24
	C: 38	C: 66.0 (9.5)	C: 7.5 (0.6)	C: 25.8 (3.9)	C: Traditional treatment		

Data are expressed as the mean (standard deviation).

S, SGLT-2i group; C, control group; NA, not applicable; HbA1c, glycated hemoglobin; BMI, body mass index; y, year; w, weeks; DXA, dual X-ray absorptiometry; BIA, bioelectrical impedance analysis; CON, control diet; OAD, oral antidiabetic agent.

### Statistical analysis

We assessed the risk of bias in each included study based on the Cochrane risk-of-bias tool, which consists of the following aspects: random sequence generation (selection bias), allocation concealment (selection bias), blinding of participants and personnel (performance bias), blinding of outcome assessment (detection bias), incomplete outcome data (attrition bias), selective reporting (reporting bias), and other biases. For each of these aspects, the assessment tool has three options: “low risk of bias,” “unclear risk of bias,” and “high risk of bias.” (41) In addition, we used the Grading of Recommendation, Assessment, Development, and Evaluation (GRADE) system to assess the quality of evidence for primary outcomes (42).

### Data synthesis

We used Review Manager 5.4 to synthesize the extracted data. Muscle-related outcome indicators were considered continuous variables and analyzed using standardized mean differences (SMD) and 95% confidence intervals (95% CI).

### Subgroup analysis and investigation of heterogeneity

Heterogeneity between the studies was assessed using the *I* ^2^ statistic. When heterogeneity was not significant (*I* ^2^ < 50%), a fixed-effects model was used to synthesize the data. When heterogeneity was significant (*I* ^2^ < 50%), a random-effects model was used.

Subgroup analyses were performed according to different definitions of muscle, the SGLT-2i measurement method, and treatment duration. In addition, sensitivity analyses were performed to assess the robustness of the meta-analysis by excluding trials with poor methodological quality (those with insufficient randomization methods and trials with selective reporting bias).

One of the subgroup analyses was performed based on different definitions of muscle: lean body mass, skeletal muscle mass, fat-free mass, and muscle mass (**Supplementary Figures 1**, **4** in **Supplementary Material 2**). Lean body mass was defined as body weight without fat minus total bone mass; skeletal muscle mass was defined as lean body mass minus connective tissue, skin, and other organ mass; and fat-free mass was defined as total body weight minus total fat mass (as defined in each original study) (40).

## Results

**Figure 1:**
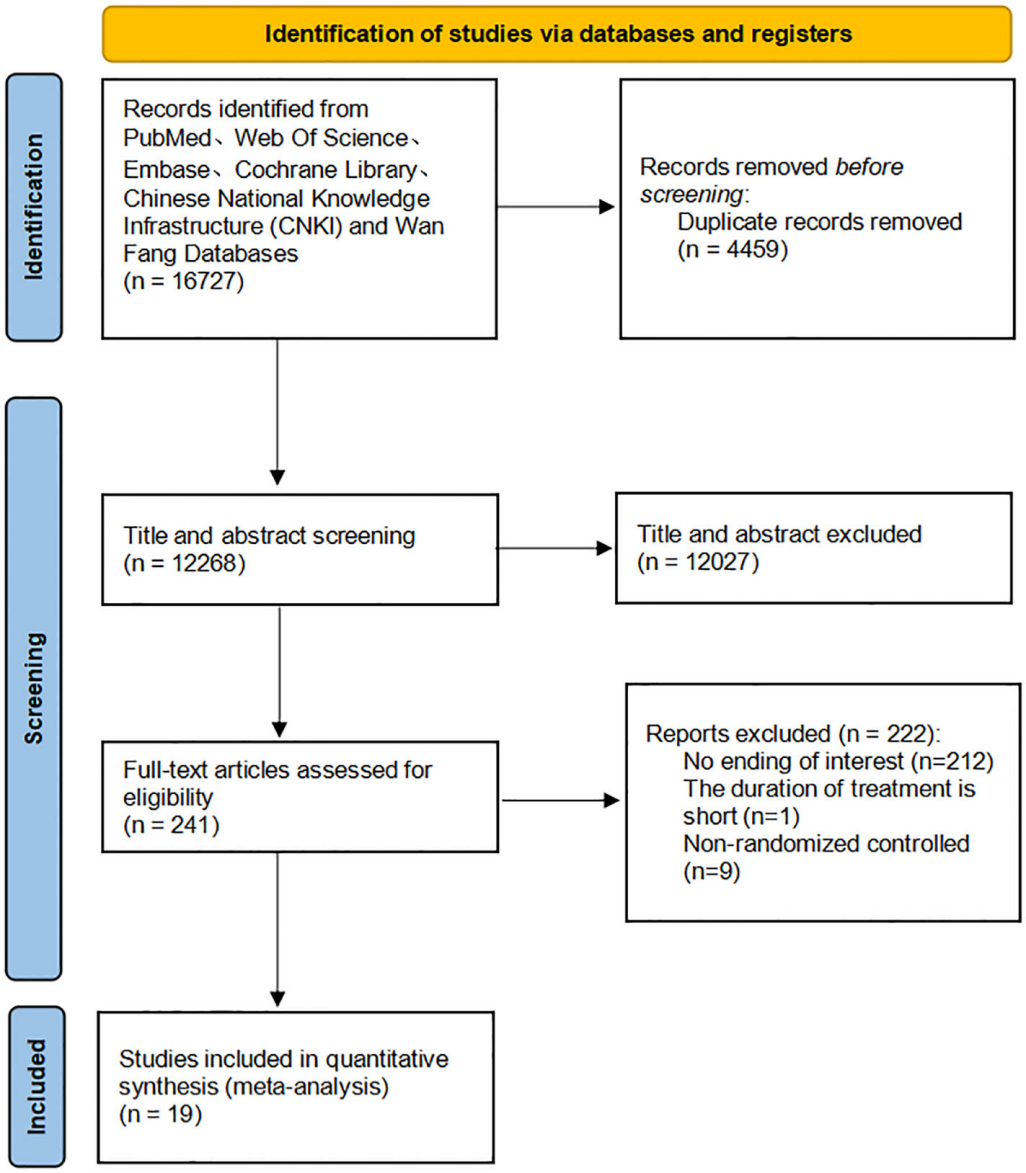
Flow chart of the included studies.

A total of 16,727 studies were identified through our search, and 19 were included (**Figure 1**).

### Study characteristics

In this systematic review, 19 RCTs involving 1,482 participants were included. Three studies (23, 26, 37) were conducted in adults with T2DM combined with nonalcoholic fatty liver disease, and the remaining 16 studies (9, 20, 24, 25, 27–36, 38, 39) were conducted in adults with T2DM. Fourteen studies (9, 23–29, 31, 32, 35, 37–39) had a treatment duration of 24 weeks, whereas the remaining five studies (20, 30, 33, 34, 36) had treatment durations beyond 24 weeks. Eight studies (20, 23, 30–34, 36) used dual X-ray absorptiometry (DXA) to measure lean body mass, six studies (9, 26, 27, 28, 29, 38) used bioelectrical impedance analysis (BIA) to measure skeletal muscle mass, one study (24) used DXA to measure lean body mass and used BIA to measure muscle mass, one study (25) used DXA to measure fat-free mass and used BIA to measure muscle mass, one study (35) used InBody to measure muscle mass, one study (37) used InBody to measure lean body mass, and one study (39) used InnerScan V with reactance technology to measure lean body mass. The sample sizes ranged from 15 to 90 patients in each study.

### Risk of bias assessment

**Figure 2:**
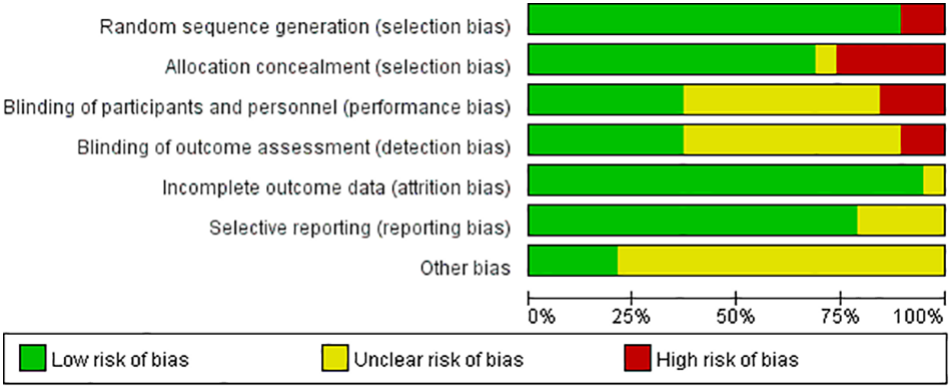
Risk of bias graph for 19 studies.

Most studies demonstrated a low or unclear risk of bias, mainly owing to a lack of blinding of the study personnel and participants (8/19, 42.1%), no blinding of the outcome assessment (10/19, 52.6%), and other unclear biases (14/19, 73.7%) (**Figure 2**).

### Effect of SGLT-2i on lean body mass

**Figure 3:**
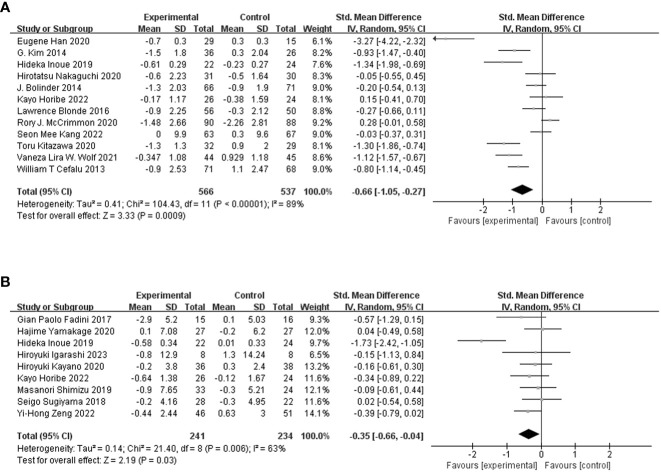
Meta-analysis of changes in lean body mass **(A)** and skeletal muscle mass **(B)** between the SGLT-2i group and the control group.

Twelve studies (20, 23–25, 28, 30–34, 36, 37) with 1,103 participants were included to compare the differences in lean body mass between the SGLT-2i and control groups. The meta-analysis results of the random-effects model showed that lean body mass was reduced by 0.66 (95% CI, −1.05, −0.27; *p* = 0.0009) in the SGLT-2i group compared to the control group (**Figure 3A**), indicating that SGLT-2i significantly reduced lean body mass in patients with T2DM. The certainty of evidence was low (**Table 2**). Because the heterogeneity was significant (I^2^ = 89%), we performed three subgroup analyses based on muscle definition, treatment duration, and measurement method (**Supplementary Figures 1, 2 and 3** in **Supplementary Material 2**). The results showed that lean body mass (SMD = −0.41; 95% CI, −0.77 to −0.04; *p* = 0.03) or fat-free mass (SMD = −0.70; 95% CI, −1.54 to 0.15; *p* = 0.11) or muscle mass (SMD = −3.27; 95% CI, −4.22 to −2.32; *p* < 0.00001) decreased, which indicated that lean body mass and muscle mass showed statistically significant reductions. We also performed subgroup analyses based on the treatment duration. The results showed that treatment duration within 24 weeks (SMD = −0.93; 95% CI, −1.60 to −0.26; *p* = 0.007) or beyond 24 weeks (SMD = −0.36; 95% CI, −0.80 to 0.07; *p* = 0.10) reduced lean body mass, and treatment duration within 24 weeks caused a statistically significant reduction in lean body mass. Our subgroup analysis also showed that compared with the control group, the lean body mass in DXA (SMD = −0.67; 95% CI, −1.12 to −0.23; *p* = 0.003) or BIA (SMD = −1.30; 95% CI, −1.86 to −0.74; *p* < 0.00001) or InBody (SMD = −0.03; 95% CI, −0.37 to 0.31; *p* = 0.86) decreased, which indicated that DXA and BIA both showed statistically significant reductions in lean body mass. Removing each study individually failed to alter the results of the meta-analysis.

**Table 2:** Summary of primary outcomes of randomized controlled trials.

Quality assessment							No of patients		Effect		Quality	Importance
No of studies	Design	Risk of bias	Inconsistency	Indirectness	Imprecision	Other considerations	SGLT-2i	Control	Relative (95% CI)	Absolute		
Lean body mass												
12	Randomized trials	No serious risk of bias	Serious^a^	No serious indirectness	Serious^b^	None	566	537	–	SMD 0.66 lower (1.05 to 0.27 lower)	⨁⨁OO LOW	Important
Skeletal muscle mass												
9	Randomized trials	No serious risk of bias	Serious^a^	No serious indirectness	Serious^b^	None	241	234	–	SMD 0.35 lower (0.66 to 0.04 lower)	⨁⨁OO LOW	Important

SMD, standardized mean differences; CI, confidence interval; NA, not applicable.

^a^ There is significant statistical heterogeneity, as indicated by a large I^2^ value.

^b^ All trials are small samples.

### Effect of SGLT-2i on skeletal muscle mass

Nine studies (9, 24–27, 29, 35, 38, 39) with 475 participants were included to compare differences in skeletal muscle mass between the SGLT-2i and control groups. The meta-analysis results of the random-effects model showed that skeletal muscle mass was reduced by 0.35 (95% CI, −0.66, −0.04; *p* = 0.03) in the group treated with SGLT-2i for T2DM compared with the control group (**Figure 3B**). The certainty of evidence was low (**Table 2**). Because the heterogeneity was significant (*I* ^2 = ^63%) and all studies including skeletal muscle mass as an outcome metric had a treatment duration of 24 weeks, we performed two subgroup analyses based on different definitions of muscle and measurement methods (**Supplementary Figures 4**, **5** in **Supplementary Material 2**). The results of subgroup analyses based on different definitions of muscle showed that skeletal muscle mass (SMD = −0.06; 95% CI, −0.31 to 0.20; *p* = 0.67) or fat-free mass (SMD = −0.57; 95% CI, −1.29 to 0.15; *p* = 0.12) or muscle mass (SMD = −0.66; 95% CI, −1.30 to −0.01; *p* = 0.05) decreased, which indicated the muscle mass showed a statistically significant reduction. Subgroup analyses were performed using this measurement method. The results showed that compared with the control group, the skeletal muscle mass in BIA (SMD = −0.40; 95% CI, −0.79 to −0.01; *p* = 0.04) or InBody (SMD = −0.15; 95% CI, −0.57 to 0.26; *p* = 0.46) decreased, which indicated that BIA showed a statistically significant reduction in skeletal muscle mass. Removing each study individually failed to alter the results of the meta-analysis.

### Adverse events

The most commonly reported adverse effects in the 19 studies were urinary tract and genital infections; however, these were not serious. No deaths were reported in any of the included studies.

## Discussion

### Summary of results

We conducted an extensive literature search and identified 19 studies (1,482 participants). Treatment with SGLT-2i in patients with T2DM resulted in statistically significant reductions in lean body mass and skeletal muscle mass compared to the controls. In a more detailed subgroup analysis of different definitions of muscle, meaningful muscle loss occurred only in lean body and muscle mass. In the subgroup analysis, lean body mass showed statistically significant reductions based on DXA and BIA measurements; skeletal muscle mass showed a statistically significant decrease in BIA measurements; and both showed a meaningless decrease in InBody measurements. In the subgroup analysis based on treatment duration, lean body mass significantly decreased at treatment duration within 24 weeks. No sources of heterogeneity were found. There was no evidence that SGLT-2i intervention led to death or serious side effects. However, the quality of evidence in the included trials was moderate to low.

### Implications for clinical research

Some studies have shown that SGLT-2i may induce or worsen muscle atrophy in patients with T2DM (14, 43–45). SGLT-2i promotes glucose excretion through urine primarily by inhibiting the reabsorption of glucose from the proximal tubules, thereby lowering blood glucose levels. However, SGLT-2i may increase energy expenditure and hypoxia in the kidney medulla. Hypoxia and a low-glucose environment can stimulate gluconeogenesis in the liver, leading to lipolysis, which reduces body fat mass (8, 46). The underlying mechanism of muscle atrophy in T2DM patients caused by SGLT-2i may be related to the fact that SGLT-2i activates gluconeogenesis and promotes lipolysis, as well as facilitates the breakdown of skeletal muscle proteins into amino acids that are supplied to the liver as substrates. However, the exact mechanism remains debatable. As the balance between vitamin D (Vit-D) and parathyroid hormone (PTH) is considered a key regulator of muscle strength, it is unclear whether SGLT-2i can disrupt this balance and cause muscle atrophy (47). Therefore, it is necessary to further explore its specific mechanisms in clinical studies.

The findings of this meta-analysis indicated that SGLT-2i resulted in statistically significant reductions in lean body mass and skeletal muscle mass. This is nearly identical to the results of a previously published study (48). That study showed meaningful and significant changes in skeletal muscle mass after SGLT-2i treatment, with a meaningless reduction in lean body mass. The latter was confirmed through our analysis. Our analysis demonstrated that following treatment with SGLT-2i, lean body mass was significantly reduced. Compared to a previous study (48), we performed more in-depth subgroup analyses based on muscle definition, measurement methods, and treatment durations. Based on the subgroup analysis, we believe that there is a clinical need to standardize the metrics that reflect muscle gain or loss so that data can be extracted and analyzed more accurately to guide clinical practice. Our data suggest that using DXA or BIA to measure lean body mass or skeletal muscle mass is more accurate than using InBody measurements. It should be noted that there are very few studies using InBody as a measurement method. Muscle loss in patients treated with SGLT-2i within 24 weeks should be closely evaluated. There is significant heterogeneity between the results of different clinical studies. Whether this heterogeneity is related to individual drug characteristics, measurement methods, or treatment cycles remains unknown and deserves further study.

Muscles and bones are closely integrated. SGLT-2i may cause muscle atrophy, which appears to be a potential factor for increased fracture risk (49–51). Researchers have suggested that the fracture risk observed in elderly patients treated with SGLT-2i does not appear to be directly related to its effect on the bone and that the effect of SGLT-2i on bone metabolism and bone turnover may be indirect (52). SGLT-2i-induced hypovolemia and hyponatremia may induce fatigue, weakness, or other psychosomatic and neurological symptoms. Muscle atrophy is a major feature of frailty (45), which can further contribute to debilitation and seriously jeopardize the health and function of the elderly. Such outcomes can lead to increased clinical adverse events such as falls, fractures, and incapacitation, severely affecting their quality of life and increasing the risk of death. Several speculations point to the possibility that fractures related to falls are secondary to factors such as weakness, upright hypotension, or postural dizziness (53, 54). Further studies are necessary to determine whether SGLT-2i-induced muscle atrophy is associated with an increased risk of fracture.

In addition, studies have shown that muscle function can be balanced and enhanced through physical activity and exercise, including resistance training, aerobic training, and whole-body vibrational therapy (55). We suggest that future clinical trials on the relationship between SGLT-2i and muscle function should consider physical exercise and exercise intensity as possible influencing factors.

### Implications for clinical practice

SGLT-2i may cause muscle atrophy, and sarcopenia is a serious consequence of muscle atrophy. Therefore, we recommend that clinicians conduct a thorough assessment of patients before drug administration, including physical indicators such as age, weight, body mass index, muscle mass, muscle strength, and muscle fat infiltration (56,) and use SGLT-2i with caution, especially in certain high-risk groups of patients with T2DM and sarcopenia (57).

The European Working Group on Sarcopenia in Older People (EWGSOP) and the Asian Working Group for Sarcopenia (AMGS) both propose muscle mass, muscle strength, and physical function as diagnostic criteria for sarcopenia (58, 59). Declines in muscle strength and body functions are a result of the loss of muscle mass and have an adverse effect on prognoses. T2DM is one of the risk factors for sarcopenia. SGLT-2i should be used in T2DM patients with attention to the risk of sarcopenia and can be used to evaluate indicators of muscle mass, muscle strength, and somatic function and thereby more comprehensively detail the possible effects of SGLT-2i treatment on muscles. In addition to DXA or BIA, diagnostic B-mode ultrasonography (60), MRI, and CT can be considered.

Several investigators have focused on the correlation between SGLT-2i and muscle atrophy. However, comprehensive and systematic research supported by sufficient data from clinical trials is still lacking. We call on researchers to focus on this issue and provide more high-quality evidence to guide future clinical practice.

### Strengths and limitations

This systematic review of the association between SGLT-2i and muscle atrophy in the treatment of T2DM provides data to answer the current question of whether SGLT-2i has harmful, insubstantial, or beneficial effects on muscles while reducing fat mass, lowering body weight, and altering the body composition of patients. We also evaluated the robustness of the meta-analysis using sensitivity analysis. Compared with a previous study (48), we went a step further and performed a subgroup analysis based on muscle definition, measurement methods, and treatment duration. We searched two Chinese databases to increase the breadth of this data.

Our review has some limitations. The results showed excessive heterogeneity, suggesting significant variability among the samples, which may have impacted the overall estimates. Subgroup analyses were performed based on different definitions of muscle, measurement methods, and treatment duration for each outcome indicator. However, subgroup analyses based on treatment duration were missing for the skeletal muscle quality group because the treatment duration of all studies using skeletal muscle quality as an outcome index was within 24 weeks. Furthermore, negligible muscle atrophy may have contributed to statistical bias. Owing to the large number of studies screened, it was uncertain whether all studies that met the criteria were included in the meta-analysis. However, we believe that these methodological limitations do not affect the overall conclusions of this meta-analysis. In addition, the studies included in this meta-analysis did not consider muscle-related indicators as primary outcomes.

## Conclusions

Our systematic review and meta-analysis suggest that treatment with SGLT-2i in patients with T2DM may lead to muscle loss. As mentioned above, this study had some limitations. To fully evaluate the possible effects of SGLT-2i treatment on the muscles of patients with T2DM, a large-sample, multicenter, and well-designed randomized controlled trial involving measures of muscle mass, muscle strength, and physical function is required.

## Data availability statement

The raw data supporting the conclusions of this article will be made available by the authors, without undue reservation.

## Author contributions

CX, YH, and MY conceived and designed the study. CX and YH drafted the manuscript. CX, YH, and CY were responsible for searching and selecting studies. CX, YH, and CY participated in data extraction and assessed the study quality. RG, ZL, and YD prepared forms and pictures and performed the statistical analyses. CX and YH wrote the first draft of the manuscript. All authors contributed to the article and approved the submitted version.
