# Supplementary material for: Relationship between sodium–glucose cotransporter-2 inhibitors and muscle atrophy in patients with type 2 diabetes mellitus: a systematic review and meta-analysis
Source: Front Endocrinol (Lausanne). 2023 Sep 15;14:1220516. doi: 10.3389/fendo.2023.1220516 (PMC10541228; doi:10.3389/fendo.2023.1220516)
Supplement: Supplementary file 1 [file DataSheet_1.pdf]

Supplementary Material 1-Search Strategies

PubMed

(((((((((((((((SGLT2 inhibitor) OR (Sodium Glucose Transporter 2 Inhibitors)) OR (SGLT-2 Inhibitors)) OR (SGLT 2 Inhibitors)) OR (SGLT2 Inhibitors)) OR (Sodium-Glucose Transporter 2 Inhibitor)) OR (Sodium Glucose Transporter 2 Inhibitor)) OR (SGLT2 Inhibitor)) OR (Inhibitor, SGLT2)) OR (Gliflozins)) OR (Gliflozin)) OR (SGLT-2 Inhibitor)) OR (Inhibitor, SGLT-2)) OR (SGLT 2 Inhibitor)) AND ((((((((((((((((((((((((((((((((Type 2 diabetes mellitus) OR (Diabetes Mellitus, Noninsulin-Dependent)) OR (Diabetes Mellitus, Ketosis-Resistant)) OR (Diabetes Mellitus, Ketosis Resistant)) OR (Ketosis-Resistant Diabetes Mellitus)) OR (Diabetes Mellitus, Non Insulin Dependent)) OR (Diabetes Mellitus, Non-Insulin-Dependent)) OR (Non-Insulin-Dependent Diabetes Mellitus)) OR (Diabetes Mellitus, Stable)) OR (Stable Diabetes Mellitus)) OR (Diabetes Mellitus, Type II)) OR (NIDDM)) OR (Diabetes Mellitus, Noninsulin Dependent)) OR (Diabetes Mellitus, Maturity-Onset)) OR (Diabetes Mellitus, Maturity Onset)) OR (Maturity-Onset Diabetes Mellitus)) OR (Maturity Onset Diabetes Mellitus)) OR (MODY)) OR (Diabetes Mellitus, Slow-Onset)) OR (Diabetes Mellitus, Slow Onset)) OR (Slow-Onset Diabetes Mellitus)) OR (Type 2 Diabetes Mellitus)) OR (Noninsulin-Dependent Diabetes Mellitus)) OR (Noninsulin Dependent Diabetes Mellitus)) OR (Maturity-Onset Diabetes)) OR (Diabetes, Maturity-Onset)) OR (Maturity Onset Diabetes)) OR (Type 2 Diabetes)) OR (Diabetes, Type 2)) OR (Diabetes Mellitus, Adult-Onset)) OR (Adult-Onset Diabetes Mellitus)) OR (Diabetes Mellitus, Adult Onset))) AND (((((((((((Randomized controlled trial) OR (Clinical Trials, Randomized)) OR (Trials, Randomized Clinical)) OR (Controlled Clinical Trials, Randomized)) OR (random allocation)) OR (double-blind)) OR (single-blind)) OR (randomly)) OR (randomized)) OR (Random*)) OR (RCT))

Web Of Science

#1 TS=(SGLT2 inhibitor OR Sodium Glucose Transporter 2 Inhibitors OR SGLT-2 Inhibitors OR SGLT 2 Inhibitors OR SGLT2 Inhibitors OR Sodium-Glucose Transporter 2 Inhibitor OR Sodium Glucose Transporter 2 Inhibitor OR SGLT2 Inhibitor OR Inhibitor, SGLT2 OR Gliflozins OR Gliflozin OR SGLT-2 Inhibitor OR Inhibitor, SGLT-2 OR SGLT 2 Inhibitor)

#2 TS=(Type 2 diabetes mellitus OR Ketosis-Resistant Diabetes Mellitus OR Stable Diabetes Mellitus OR NIDDM OR Maturity Onset Diabetes Mellitus OR MODY OR Slow-Onset Diabetes Mellitus OR Diabetes,Maturity-Onset OR Maturity Onset Diabetes OR Diabetes,Type 2 OR Adult-Onset Diabetes Mellitus)

#3 TS=(Randomized controlled trial OR Clinical Trials, Randomized OR Trials, Randomized Clinical OR Controlled Clinical Trials, Randomized OR random allocation OR double-blind OR single-blind OR randomly OR randomized OR Random* OR RCT)

#4 #1 AND #2 AND #3

Embase

#1 'SGLT2 inhibitor' OR 'Sodium Glucose Transporter 2 Inhibitors' OR 'SGLT-2 Inhibitors' OR 'SGLT 2 Inhibitors' OR 'Sodium-Glucose Transporter 2 Inhibitor' OR

'Sodium Glucose Transporter 2 Inhibitor' OR 'SGLT2 Inhibitor' OR 'Inhibitor, SGLT2 OR Gliflozins' OR 'Gliflozin' OR 'SGLT-2 Inhibitor' OR 'Inhibitor, SGLT-2' OR 'SGLT 2 Inhibitor'

#2 'Type 2 diabetes mellitus' OR 'Diabetes Mellitus*' OR 'Ketosis-Resistant Diabetes Mellitus' OR 'Stable Diabetes Mellitus' OR 'NIDDM' OR 'Maturity-Onset Diabetes Mellitus' OR 'Maturity Onset Diabetes Mellitus' OR 'MODY' OR 'Slow-Onset Diabetes Mellitus' OR 'Noninsulin Dependent Diabetes Mellitus' OR 'Maturity-Onset Diabetes' OR 'Diabetes, Maturity-Onset' OR 'Maturity Onset Diabetes' OR 'Type 2 Diabetes' OR 'Diabetes, Type 2' OR 'Adult-Onset Diabetes Mellitus' OR 'Type 2 diabetes mellitus'

#3 'Randomized controlled trial' OR 'Clinical Trials, Randomized' OR 'Trials, Randomized Clinical' OR 'Controlled Clinical Trials, Randomized' OR 'random allocation' OR 'double-blind' OR 'single-blind' OR 'randomly' OR 'randomized' OR 'Random*' OR 'RCT'

#4 #1 AND #2 AND #3

Cochrane Library

#1 SGLT2 inhibitor OR Sodium Glucose Transporter 2 Inhibitors OR SGLT-2 Inhibitors OR SGLT 2 Inhibitors OR SGLT2 Inhibitors OR Sodium-Glucose Transporter 2 Inhibitor OR Sodium Glucose Transporter 2 Inhibitor OR SGLT2 Inhibitor OR Inhibitor, SGLT2 OR Gliflozins OR Gliflozin OR SGLT-2 Inhibitor OR Inhibitor, SGLT-2 OR SGLT 2 Inhibitor

#2 Type 2 diabetes mellitus OR Stable Diabetes Mellitus OR NIDDM OR Maturity-Onset Diabetes Mellitus OR Maturity Onset Diabetes Mellitus OR MODY OR Slow-Onset Diabetes Mellitus OR Noninsulin Dependent Diabetes Mellitus OR Maturity-Onset Diabetes OR Diabetes, Maturity-Onset OR Maturity Onset Diabetes OR Type 2 Diabetes OR Diabetes, Type 2 OR Adult-Onset Diabetes Mellitus OR Type 2 diabetes mellitus

#3 Randomized controlled trial OR Clinical Trials, Randomized OR Trials, Randomized Clinical OR Controlled Clinical Trials, Randomized OR random allocation OR double-blind OR single-blind OR randomly OR randomized OR Random* OR RCT

#4 #1 AND #2 AND #3

Chinese National Knowledge Infrastructure Database (CNKI) & Wanfang Database

SGLT2 抑制剂 OR SGLT2i OR SGLT-2i OR 钠葡萄糖共转运蛋白 2 抑制剂 OR 钠-葡萄糖共转运蛋白 2 抑制剂 and 2 型糖尿病 OR 二型糖尿病 OR T2DM OR 成人发病型糖尿病 OR NIDDM OR 非胰岛素依赖型糖尿病 and 随机对照试验 OR 随机 OR 对照 OR RCT
