# Supplementary material for: Relationship between sodium–glucose cotransporter-2 inhibitors and muscle atrophy in patients with type 2 diabetes mellitus: a systematic review and meta-analysis
Source: Front Endocrinol (Lausanne). 2023 Sep 15;14:1220516. doi: 10.3389/fendo.2023.1220516 (PMC10541228; doi:10.3389/fendo.2023.1220516)
Supplement: Supplementary file 2 [file DataSheet_2.pdf]

# Supplementary Material 2-Subgroup analysis

| Study or Subgroup | Experimental Mean | Experimental SD | Experimental Total | Control Mean | Control SD | Control Total | Weight | Std. Mean Difference IV, Random, 95% CI |
|---|---|---|---|---|---|---|---|---|
| **1.1.1 lean body mass** | | | | | | | | |
| Hideka Inoue 2019 | -0.61 | 0.29 | 22 | -0.23 | 0.27 | 24 | 7.6% | -1.34 [-1.98, -0.69] |
| Hirotatsu Nakaguchi 2020 | -0.6 | 2.23 | 31 | -0.5 | 1.64 | 30 | 8.3% | -0.05 [-0.55, 0.45] |
| J. Bolinder 2014 | -1.3 | 2.03 | 66 | -0.9 | 1.9 | 71 | 9.0% | -0.20 [-0.54, 0.13] |
| Lawrence Blonde 2016 | -0.9 | 2.25 | 56 | -0.3 | 2.12 | 50 | 8.8% | -0.27 [-0.66, 0.11] |
| Rory J. McCrimmon 2020 | -1.48 | 2.66 | 90 | -2.26 | 2.81 | 88 | 9.2% | 0.28 [-0.01, 0.58] |
| Seon Mee Kang 2022 | 0 | 9.9 | 63 | 0.3 | 9.6 | 67 | 9.0% | -0.03 [-0.37, 0.31] |
| Vaneza Lira W. Wolf 2021 | -0.347 | 1.08 | 44 | 0.929 | 1.18 | 45 | 8.6% | -1.12 [-1.57, -0.67] |
| William T Cefalu 2013 | -0.9 | 2.53 | 71 | 1.1 | 2.47 | 68 | 9.0% | -0.80 [-1.14, -0.45] |
| **Subtotal (95% CI)** | | | **443** | | | **443** | **69.5%** | **-0.41 [-0.77, -0.04]** |
| Heterogeneity: Tau² = 0.23; Chi² = 49.22, df = 7 (P < 0.00001); I² = 86% | | | | | | | | |
| Test for overall effect: Z = 2.20 (P = 0.03) | | | | | | | | |
| **1.1.2 fat free mass** | | | | | | | | |
| G. Kim 2014 | -1.5 | 1.8 | 36 | 0.3 | 2.04 | 26 | 8.2% | -0.93 [-1.47, -0.40] |
| Kayo Horibe 2022 | -0.17 | 1.17 | 26 | -0.38 | 1.59 | 24 | 8.1% | 0.15 [-0.41, 0.70] |
| Toru Kitazawa 2020 | -1.3 | 1.3 | 32 | 0.9 | 2 | 29 | 8.1% | -1.30 [-1.86, -0.74] |
| **Subtotal (95% CI)** | | | **94** | | | **79** | **24.3%** | **-0.70 [-1.54, 0.15]** |
| Heterogeneity: Tau² = 0.48; Chi² = 14.19, df = 2 (P = 0.0008); I² = 86% | | | | | | | | |
| Test for overall effect: Z = 1.62 (P = 0.11) | | | | | | | | |
| **1.1.3 muscle mass** | | | | | | | | |
| Eugene Han 2020 | -0.7 | 0.3 | 29 | 0.3 | 0.3 | 15 | 6.1% | -3.27 [-4.22, -2.32] |
| **Subtotal (95% CI)** | | | **29** | | | **15** | **6.1%** | **-3.27 [-4.22, -2.32]** |
| Heterogeneity: Not applicable | | | | | | | | |
| Test for overall effect: Z = 6.75 (P < 0.00001) | | | | | | | | |
| **Total (95% CI)** | | | **566** | | | **537** | **100.0%** | **-0.66 [-1.05, -0.27]** |
| Heterogeneity: Tau² = 0.41; Chi² = 104.43, df = 11 (P < 0.00001); I² = 89% | | | | | | | | |
| Test for overall effect: Z = 3.33 (P = 0.0009) | | | | | | | | |
| Test for subgroup differences: Chi² = 30.45, df = 2 (P < 0.00001), I² = 93.4% | | | | | | | | |

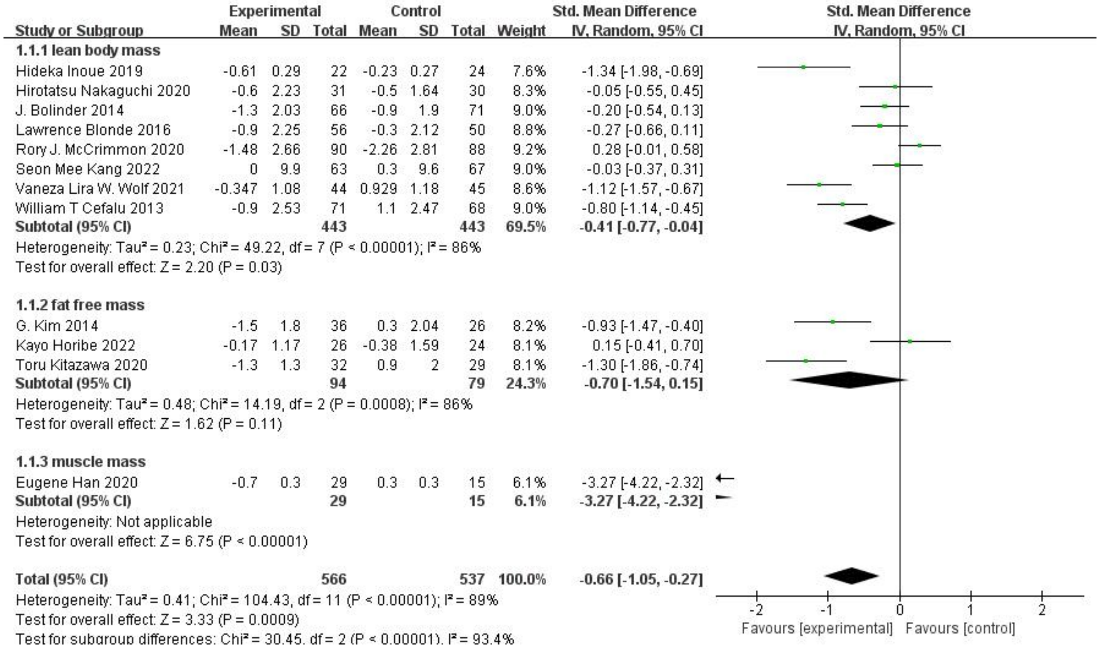


**Figure 1.** Subgroup analysis of changes in lean body mass between the SGLT-2i group and the control group - muscle definition

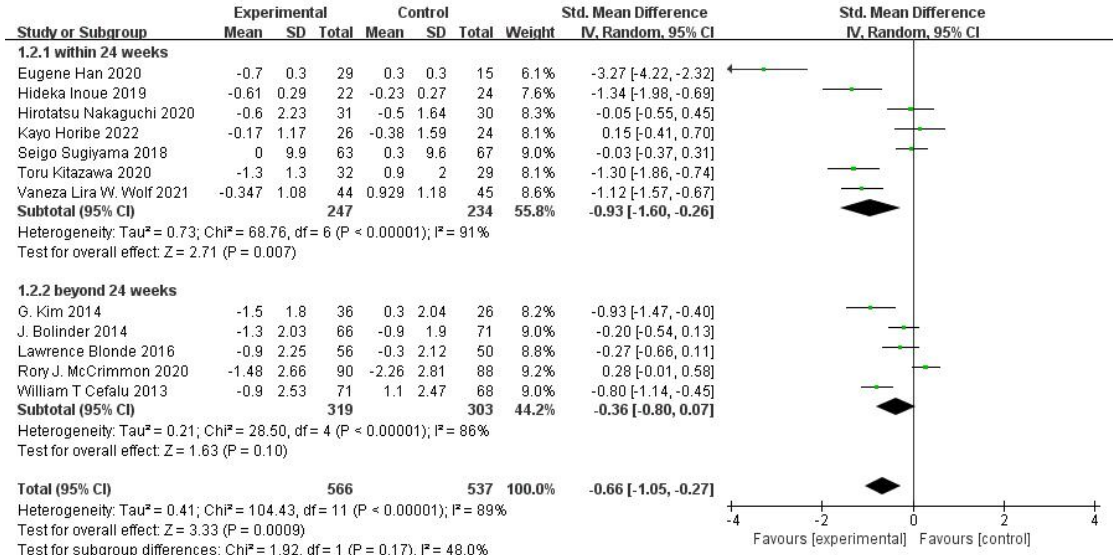


**Figure 2.** Subgroup analysis of changes in lean body mass between the SGLT-2i group and the control group - treatment duration

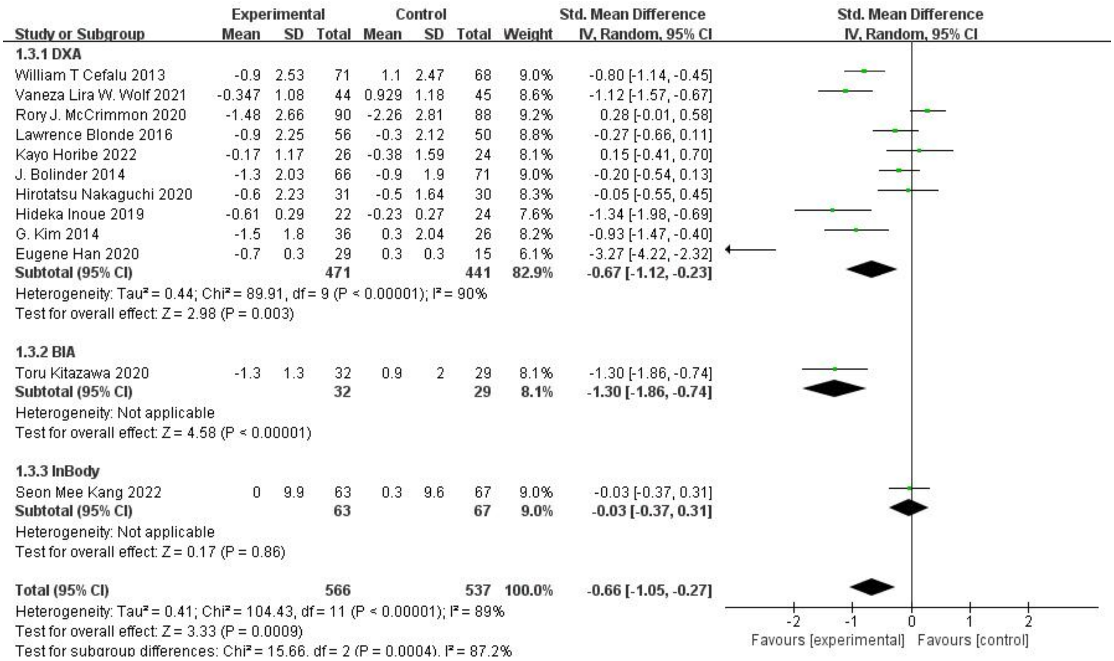


**Figure 3.** Subgroup analysis of changes in lean body mass between the SGLT-2i group and the control group - measurement method

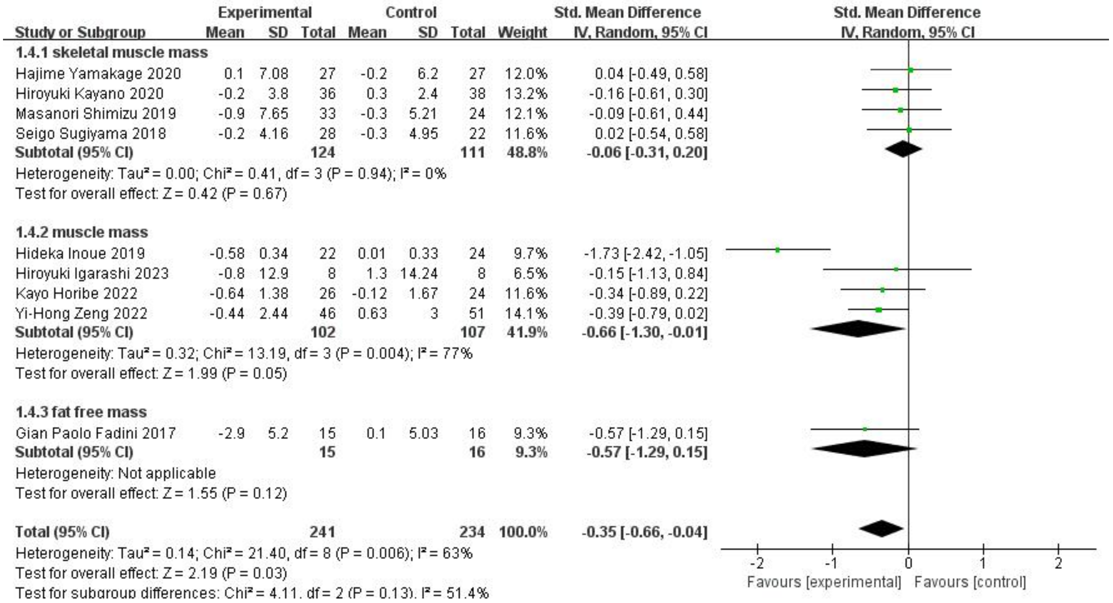


**Figure 4.** Subgroup analysis of changes in skeletal muscle mass between the SGLT-2i group and the control group - muscle definition

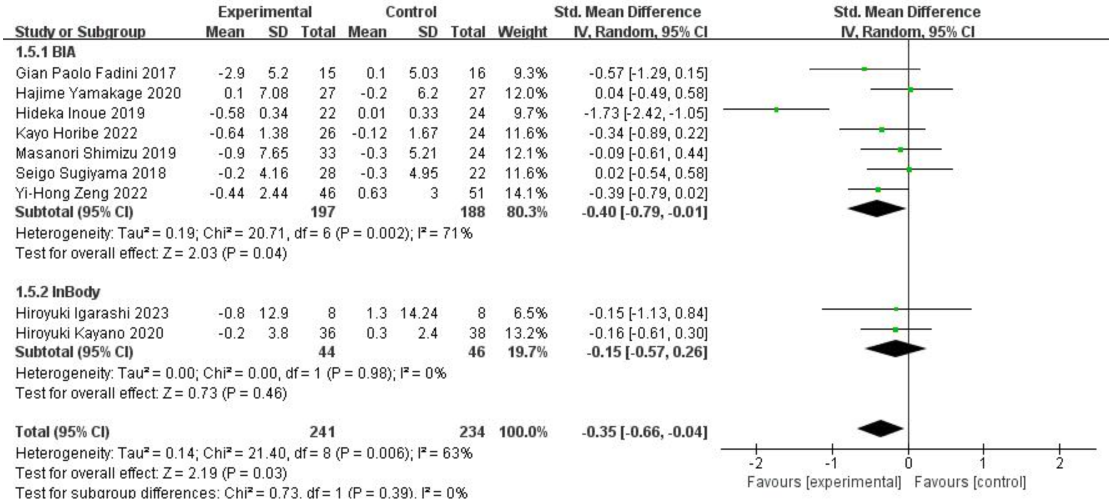


**Figure 5.** Subgroup analysis of changes in skeletal muscle mass between the SGLT-2i group and the control group - measurement method
